# Utilization of Sugarcane Habitat by Feral Pig (*Sus scrofa*) in Northern Tropical Queensland: Evidence from the Stable Isotope Composition of Hair

**DOI:** 10.1371/journal.pone.0043538

**Published:** 2012-09-05

**Authors:** Christopher M. Wurster, Jack Robertson, David A. Westcott, Bart Dryden, Antoine Zazzo, Michael I. Bird

**Affiliations:** 1 School of Earth and Environmental Sciences and Centre for Tropical Environmental and Sustainability Science, James Cook University, Cairns, Queensland, Australia; 2 Commonwealth Scientific and Industrial Research Organization (CSIRO) Ecosystem Sciences, Atherton, Queensland, Australia; 3 Terrain Natural Resource Management, Innisfail, Queensland, Australia; 4 Centre National de la Recherche Scientifique (CNRS) UMR 7209 and Muséum National d'Histoire Naturelle, Paris, France; University of Plymouth, United Kingdom

## Abstract

Feral pigs (*Sus scrofa*) are an invasive species that disrupt ecosystem functioning throughout their introduced range. In tropical environments, feral pigs are associated with predation and displacement of endangered species, modification of habitat, and act as a vector for the spread of exotic vegetation and disease. Across many parts of their introduced range, the diet of feral pigs is poorly known. Although the remote location and difficult terrain of far north Queensland makes observing feral pig behavior difficult, feral pigs are perceived to seek refuge in World Heritage tropical rainforests and seasonally ‘crop raid’ into lowland sugarcane crops. Thus, identifying how feral pigs are using different components of the landscape is important to the design of management strategies. We used the stable isotope composition of captured feral pigs to determine the extent of rainforest and sugarcane habitat usage. Recently grown hair (basal hair) from feral pigs captured in remote rainforest indicated pigs met their dietary needs solely within this habitat. Stable carbon and nitrogen isotope values of basal hair from feral pigs captured near sugarcane plantations were more variable, with some individuals estimated to consume over 85% of their diet within a sugarcane habitat, while a few consumed as much as 90% of their diet from adjacent forested environments. We estimated whether feral pigs switch habitats by sequentially sampling *δ* ^13^C and *δ* ^15^N values of long tail hair from a subset of seven captured animals, and demonstrate that four of these individuals moved between habitats. Our results indicate that feral pigs utilize both sugarcane and forest habitats, and can switch between these resources.

## Introduction

Invasive feral pigs (*Sus scrofa*) have deleterious effects on ecosystem functioning across their introduced range [1]. Direct negative impacts have been attributed to feral pigs including predation and displacement of endangered species, modification of habitat, and serving as a vector for the spread of exotic vegetation and endemic disease, with notable potential for the spread of exotic disease [2]. Feral pig introductions have been found to indirectly impact food webs and cause a decline in native predator abundance [3]. Australia has seen a wide range of programs developed to control feral pigs but these have generally been piecemeal with little evaluation of their effectiveness [2].

In the Australian tropics, feral pigs have been implicated in increased erosion, lowered water quality, shifting litter composition, as well as the modification of biogeochemical cycles, soil invertebrate and seed bank composition, and species successional patterns [4]–[6]. Diggings are the most visible impact of feral pigs in the World Heritage listed tropical rainforests of northern Queensland [4]–[6]. Although large-scale and long-term ecological impacts of these diggings have not been established, severe impacts on small-scale microhabitats within the rainforest have been suggested [5]. Seedling survival increased in feral pig exclosures, however pig diggings did not affect earthworm biomass, litter composition, root mass, or soil moisture levels [5]. Properly assessing the role of feral pig activity on their surrounding environment requires an understanding of their diet, however, this aspect of pig ecology is largely unknown in tropical northern Queensland. For example, although feral pigs were reported to be dependent on earthworms near Cape Tribulation [7], this was discounted in a subsequent study near Cardwell, only 150 km to the south [5]. Laurance and Harrington [4] hypothesized that feral pigs may feed on fungal sporocarps thereby competing with endangered northern bettongs, and recommended feral pig diet become a research priority.

Questions about the diet of feral pigs in north Tropical Queensland also encompass their use of agricultural land. Economically, feral pigs impact agriculture, generating more than $100 million annually in damage in Queensland [8], which compares to $800 million in the United States [1]. The general perception in the farming community is that ‘crop raiding’ occurs where feral pigs move out of World Heritage rainforests seasonally to raid crops [9]. However, this contention was not supported by telemetry that showed feral pigs to be sedentary, with home ranges of approximately eight km^2^ [9]. This raises the issue of how management of feral pigs should be conducted. Very different spatial strategies are implied for management if the pigs that cause damage move out of conservation reserves to forage in farmland than if pigs are sedentary. A central issue is whether Mitchell et al.'s [9] observations of relatively sedentary animals hold across complex landscapes with both agricultural and natural vegetation types on a seasonal basis.

The stable isotope composition of feral pig hair offers a method for quantifying feral pig diet and habitat use, and sequential analyses of longer tail hair can resolve temporal changes in resource utilization. The stable isotope compositions of animal tissue reflect dietary values with varying degrees of trophic enrichment (e.g. [10]–[12]). In particular, stable carbon and nitrogen isotope compositions are linked to biochemical processes during carbon fixation in plants, and habitat use can thereby be directly linked to the animal (e.g. [13]–[15]). Like other biogeochemical markers, stable isotopes are particularly useful for studying animal movements on various scales because they do not require marking or recapturing individuals and they provide time-integrated information that can be linked directly to geographical regions under favorable circumstances [13].

Different physiological pathways of carbon fixation in plants yield differing stable carbon isotope ratios [16]. Stable carbon isotope values of C_3_ plants are lower than C_4_ vegetation due to different enzymatic discriminations of the heavy isotope through the carbon fixing pathways. Woody vegetation (rainforest) almost exclusively uses C_3_ photosynthesis [17], [18]. Because sugarcane is a C_4_ plant, a diet based in this habitat is readily discernable from a diet based on rainforest habitat. Analysing the stable isotope composition of hair offers further advantages as it is non-invasive, and sequential sampling represents changing dietary habits with time, so seasonal changes in habitat use can be identified [11], [14], [19].

In this study, we collected recently grown basal hair (3 mm closest to the skin) from 41 feral pigs in north tropical Queensland captured from rainforest and sugarcane habitats to identify resource utilization and dietary preferences. We assumed that basal hair from feral pigs captured in remote rainforest locations would indicate a diet based exclusively on C_3_ resources in order to compare diet-hair fractionation with published values. We then tested the hypothesis that pigs closer to sugarcane habitat are more likely to use sugarcane derived-resources. To examine the possibility that pigs move seasonally from rainforest to feed in sugarcane, we sampled tail hair sequentially from a subset of seven individuals.

## Results

### Stable isotope composition of basal hair and soils

**Figure 1:**
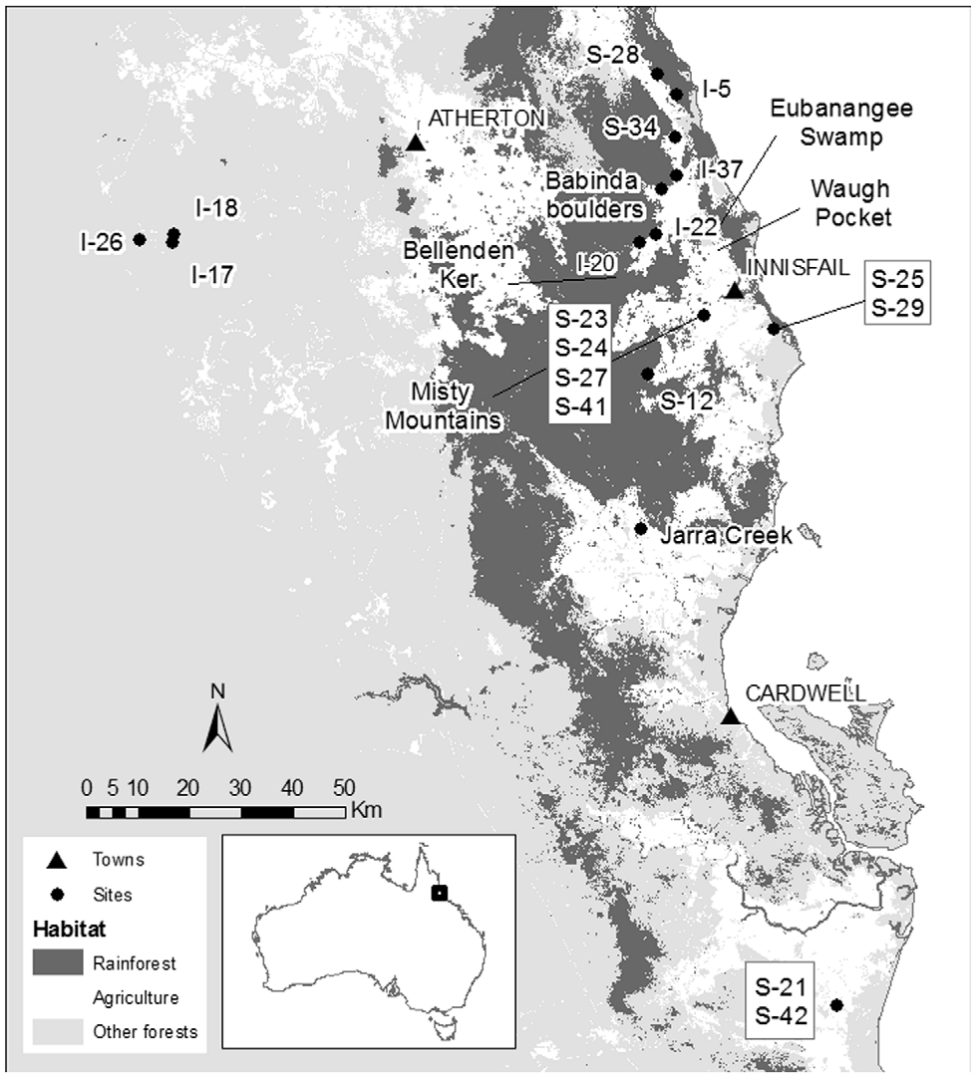
Study Area showing capture sites of feral pigs in this study. Feral pigs captured in cane plantations are denoted with an S, while those from interface habitats are denoted with an I. Pigs captured in Jarra Creek, Misty Mountains, and Babinda Boulders are deemed captured in rainforest.

**Figure 2:**
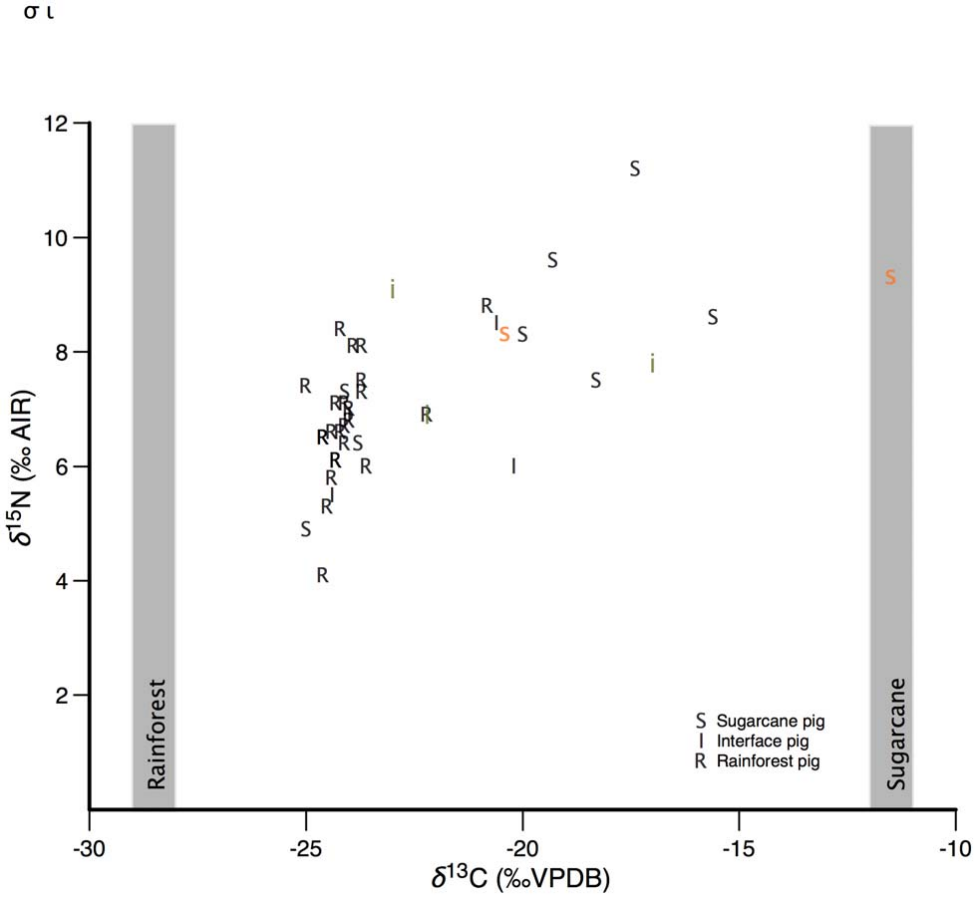
δ^13^C and δ^15^N values of feral pig base hair. The stable isotope composition of base hair were taken from 41 feral pigs captured at Rainforest (R), Savanna (S), or Interface environments (I). Sugarcane and Rainforest *δ* ^13^C and *δ* ^15^N value endmembers are plotted for comparison. A lowercase letter (s, i) indicates that the site contains ready access to native C_4_ vegetation.

**Figure 3:**
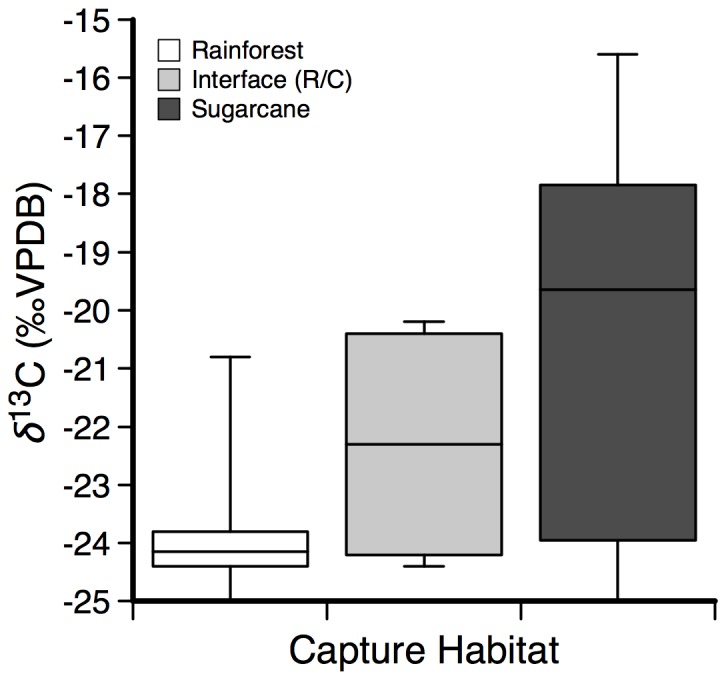
Box and whisker plot showing mean δ^13^C values of base hair.

Pigs were captured from various locations in the central section of the Wet Tropics Bioregion of northeastern Australia (Figure 1). A substantial range in both *δ* ^13^C and *δ* ^15^N values were determined for 41 samples of basal hair analyzed in this study indicating that pigs utilized both rainforest and sugarcane habitat, four of these captured pigs had ready access to native C_4_ grass understory, but the remainder would have had access to C_4_ habitat only through sugarcane plantations (Figure 2). We found no significant differences between male and female stable isotope compositions of hair (*δ* ^13^C value: ANOVA *F_1,39_* = 0.04, *p*>0.5; *δ* ^15^N value: ANOVA *F_1,39_* = 0.87, *p* = 0.36), although there were significant differences in the stable isotope composition of hair between pigs captured in either rainforest, interface, or cane habitat (*δ* ^13^C value: ANOVA *F_2,38_* = 12.92, *p*<0.001; Figure 3; *δ* ^15^N value: ANOVA *F_2,38_* = 3.9, *p* = 0.03). The lowest *δ* ^13^C value measured for pig hair was −25‰, consistent with an approximately 97% C_3_ diet assuming a +3‰ enrichment factor and basline values of −28.5‰, and −12.2‰ for rainforest and sugarcane, respectively (see methods). *δ* ^13^C values of basal hair from pigs captured in rainforest habitat displayed a reduced range compared to pigs captured from interface or sugarcane habitats, with only one significant outlier (Figure 3). *δ* ^15^N values of hair range from +4.1 to +11.2‰ and cannot be simply attributed to habitat due to variable *δ* ^15^N in habitats and potential diet. Nonetheless, there is a significant relationship between *δ* ^13^C values and *δ* ^15^N values within the cane capture group (*r^2^* = 0.49, *p* = 0.025), and all groups combined (*r^2^* = 0.40 *p*<0.001), suggesting that cane habitat tended to have higher baseline *δ* ^15^N values. Soils from a range of local cane sugar plantations were found to vary from +1.5 to +7.7‰ (this study), with over half having *δ* ^15^N values over +6.4‰.

### Sequential δ^13^C and δ^15^N values of hair

**Figure 4:**
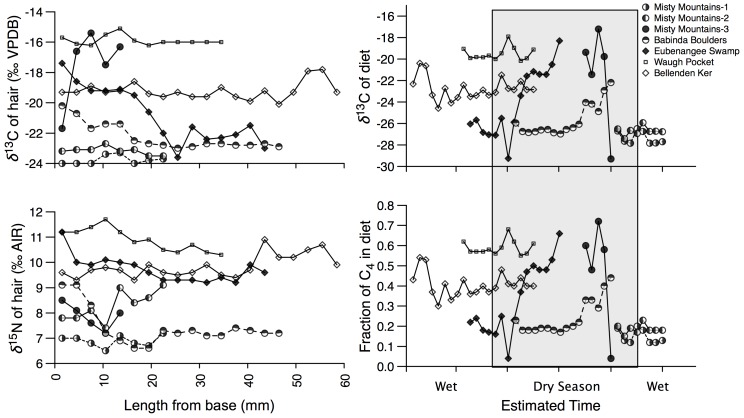
Sequential *δ* ^13^C and *δ* ^15^N values of longer tail hair and derived information. *δ* ^13^C (A) and *δ* ^15^N (B) values for seven feral pigs captured from different environments (rainforest, interface, and sugarcane). *δ* ^13^C values of sequential feral pig hair were used to estimate *δ* ^13^C dietary values (C), and estimated fraction of C_4_ in the diet (D). Feral pigs from the Misty Mountains (MM1-50 Kg male, MM2-80 Kg male, MM3-30 Kg female) and Babinda Boulders (60 Kg male) were captured in rainforest environments. Feral pigs from Eubenangee Swamp (60 Kg male), Waugh Pocket (100 Kg male), and Bellenden Ker (40 Kg female) were captured in Sugarcane plantations. The dry season is shaded.

**Figure 5:**
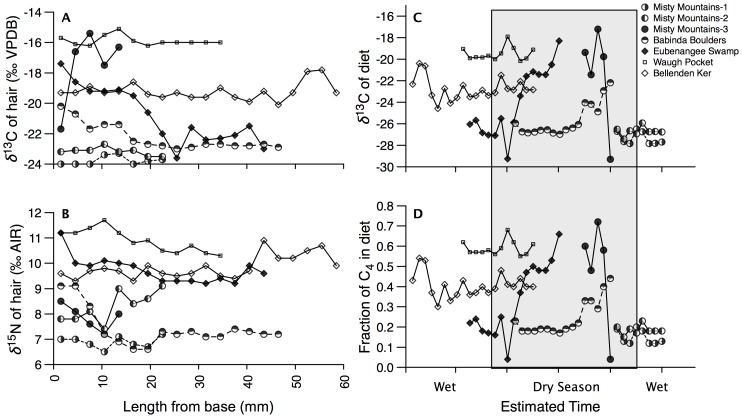
Plot of δ^13^C vs. δ^15^N values of sequential feral pig hair from seven individuals. Specimen legend as in Figure 4.

Seven individuals from representative habitat collection sites were selected for sequential analysis of tail hair (see methods). Sequential *δ* ^13^C and *δ* ^15^N values exhibited similar variability to that of individual hairs measured from 41 individuals and ranged from −24.0 to −15.1‰ (Figure 4a). Three individuals exhibited δ^13^C values at least as high as −18‰, and two individuals varied over 6‰ within a single tail hair. Our time-resolved record of temporal variation in the *δ* ^13^C values of diet derived from a three-pool mixing model and assuming a hair growth rate of 0.4 mm/day [20], was able to distinguish temporal change in the proportion of C_4_ (sugarcane)-derived carbon in the diet of the animals (Figure 4b). A 3-mm section of hair was equivalent to ∼7.5 days using the assumed growth rate, and we modeled minimum dietary value of −29.8‰, and maximum value of −17.0‰. The results suggest a dietary shift in *δ* ^13^C values of over 10‰ for two individuals, while another two individuals displayed shifts over 4.5‰, with the remaining animals showing no discernable change in diet. Sequential *δ* ^15^N values were less variant than *δ* ^13^C values, with at most a ∼2.5‰ range within one individual (Figure 4). Nonetheless, there was a significant correlation between *δ* ^13^C and *δ* ^15^N values of sequential hair samples (*r^2^* = 0.54, *p*>0.001), which includes the notable exception of a 30-kg female captured from the Misty Mountains that exhibited relatively invariant *δ* ^15^N values (Figure 5). In general, higher *δ* ^15^N values are associated with an increased dependency on a C_4_-based (sugarcane) diet.

## Discussion

The stable isotope composition of basal hair demonstrated that a significant proportion of the pigs sampled fed directly from a C_4_ habitat during at least part of the year. Using a simple mixing model with endmembers of −12.2‰ representing an entirely C_4_-based diet and −28.5‰ representing an entirely C_3_ rainforest-based diet, the stable isotope composition of basal hair showed that feral pigs utilized between 3 and 86% C_4_-based resources in their diet. Pigs captured in rainforest locations far from sugarcane plantations yielded the lowest variation, and had consistently over 90% C_3_ resource utilization, while pigs caught in sugarcane plantations yielded the greatest range in C_4_ -resource utilization (3–86%).

While the general pattern of recent resource exploitation (as identified by basal hair) by the sampled pigs matched the pattern predicted, there were individuals within both rainforest and sugarcane contexts that showed greater than expected utilization of either C_4_ or C_3_ resources, respectively. There are several possible explanations for this variation. The first is that neither forest nor agricultural landscapes are homogenous; consequently some small percentage of both C_3_ and C_4_ resources will be found in all habitats and, furthermore, individuals that utilize a small component of the landscape more intensively may show a different pattern of resource use from that expected from the more general characteristics of the landscape. Most feral pigs captured in a rainforest habitat far from sugarcane or savanna environments had a over 95% C_3_-diet, although one individual captured within a rainforest habitat utilized up to 29% C_4_-based resources. This pig is notably the largest in our sample set at 120 kg, and may have been a recent migrant that had not equilibrated with its habitat, or this pig may have targeted cleared powerline corridors or along roads within forested landscapes that contain significant C_4_ biomass. Feral pigs caught within sugarcane habitat yielded much more variable results, ranging from ∼10 to 85% C_4_-based diet. In sugarcane-dominated parts of the landscape, native vegetation components are common and sometimes locally extensive. Differential use of these more forested habitats is a potential explanation for the less than 20% C_4_-based resource we estimate for three of the ten pigs captured in these habitats. Finally, it must be realized that vegetation and dietary resources have inherent variability in their stable isotope compositions. For example, worldwide C_3_ vegetation ranges from −43 to −20‰ VPDB [21], and although variation of local tropical rainforest vegetation is much reduced, values up to −27‰ VPDB have been measured.

We can reasonably attribute the relatively high *δ* ^15^N values in some pig hairs to high baseline *δ* ^15^N values of cane plantations. Soil from eight local cane plantations had *δ* ^15^N values that ranged from +1.5 to +7.7‰; over half of these soils had *δ* ^15^N values over +6.4‰, which were similar to relatively high local cane *δ* ^15^N values between 6–8‰ from an earlier study [22]. These relatively high *δ* ^15^N values are likely due to the addition of mill mud, a manufactured byproduct of sugar extraction reapplied to the soil as fertilizer. This suggests that soil *δ* ^15^N values from different plantations are variable, but often higher than local rainforest soils in north Queensland (Bird-unpublished data). Interestingly, *δ* ^13^C and *δ* ^15^N values of snakeskin, composed dominantly of keratin (as is hair), from a taipan caught on a cane plantation exemplifies this pattern. Taipan's dominantly consume rodents and are often resident in cane plantations. The skin was found to have a *δ* ^13^C value of −11.7‰, and a *δ* ^15^N value of +11.8‰. These values might serve as an approximation of the stable isotope composition of a top predator within cane habitat. Notably, several feral pigs had *δ* ^13^C and *δ* ^15^N values similar to those of the taipan.

Sequential analysis of the stable isotope composition of tail hair indicated that pigs were as likely to move between habitats as they were to remain sedentary. A feral pig caught in Eubanangee swamp sugar cane plantation was a recent migrant, having subsided on a C_3_ (rainforest)-based diet ∼45 days prior to capture, while consuming ∼60% C_4_ –based diet (sugarcane) within the week preceding capture. Another feral pig caught in the Misty Mountain rainforest displays the nearly opposite pattern, changing from an over 50% C_4_-based diet (sugarcane) to that based entirely on C_3_ vegetation within the final week. In contrast, two additional male individuals captured in the Misty Mountain region were sedentary, maintaining a consistent C_3_–based diet for at least the previous 40 days.

Two individuals captured in a rainforest habitat displayed δ^13^C values indicative of some incorporation of C_4_-diet (Figure 4). A feral pig captured in Babinda Boulders consumed over 90% C_3_-based diet until the final days prior to capture when this individual increased its consumption of C_4_ to nearly 40%. A second individual caught in a sugarcane plantation near Mount Bellenden Ker consumed a reasonably consistent 35% C_4_ –based diet for at least several months prior to capture. One individual caught in Waugh Pocket sugarcane habitat, displayed high *δ* ^13^C values indicative of a consistent 50–60% C_4_–based diet for approximately three months of this feral pig's life prior to capture. *δ* ^13^C and *δ* ^15^N values were positively correlated for most samples from most individuals, with the exception of one individual captured in a rainforest. Clearly, this individual was a recent migrant to the area, and the consistently low *δ* ^15^N values indicate that it may have migrated from a cane plantation with lower baseline *δ* ^15^N values. The results demonstrate that feral pigs can utilize a substantial amount of C_4_-based resources in their diet, and over half of our sampled individuals moved from one habitat to another during the time interval represented in their tail hair. Across most of this region, a C_4_-based diet is highly likely to indicate that foraging took place within sugarcane plantations. This observation suggests that though some pigs are utilizing both native vegetation and agricultural lands, others are resident primarily or solely within one context or the other. As a consequence, while management of pigs on the interface between these habitats, and conservation reserves and agricultural land, may control the impact of the pigs resident in these areas, conservation reserves are not the sole source of pigs encountered in the agricultural lands. Feral pig abatement programs aimed at ecological outcomes will need to focus on populations located in remote rainforest habitats in addition to populations utilizing interface habitat. Feral pig management must therefore be applied across the entire landscape for it to be effective. We show the possibility of pig movement across the landscape, but more work will be required to determine population aspects of habitat utilization.

Our stable isotope results on hair show that a significant proportion of feral pigs sampled in tropical far north Queensland utilize dietary resources found in sugarcane habitat. The results also demonstrate that half of our sampled individuals moved between rainforest and sugarcane habitats in the months leading up to their capture. As expected, recent hair growth from pigs captured in a rainforest habitat far from savanna or sugarcane habitat yielded almost exclusively C_3_ dietary values, which in turn supported our use of a +3.0‰ enrichment factor. Only one ‘rainforest’ individual out of 24 yielded any significant incorporation of a C_4_-based diet. δ^13^C values of hair from feral pigs captured in ‘interface’ or ‘sugarcane’ habitats were much more variable, indicating a range of dietary preferences, and as much as an 85% dependency on C_4_-dominated habitat. We also demonstrate the potential for increased geospatial and temporal resolution using stable isotope composition of hair. The strong rainfall gradient of the region likely leads to additional potential. Additional measurement of other biogeochemical markers (e.g., sulphur, hydrogen, and/or oxygen isotope composition) would likely increase geospatial tracking potential for movements of individual pigs. Moreover, additional information on the dietary items consumed by feral pigs would better inform the interpretation of *δ* ^13^C and *δ* ^15^N values analyzed from hair as these are a function of assimilated food which may vary because of differences in diet.

The stable isotope composition of hair from feral pigs is a powerful proxy for determination of pig diet and habitat utilization in the tropics, where significant areas of C_4_ vegetation are found. Future experimental studies will enable more confidence to be placed in model parameters and thus upon isotope-based dietary estimates. Experimental determination of feral pig hair growth rates will enable us to refine our temporal and spatial understanding of feral pig movement in tropical terrains, where biogeochemical markers have distinct advantages over more traditional extrinsic markers of animal movement. Critically, a further study is required to address how frequently feral pigs migrate over a full season. More individuals should be targeted for sequential isotope measurements on pigs captured during the same season from different environments.

## Materials and Methods

### Study area

The study was conducted in the central section of the Wet Tropics Bioregion of northeastern Australia (Figure 1). In this region, marked topographical relief immediately adjacent to the coast produces a strong rainfall gradient with annual rainfall varying from c. 1200 mm to 7500 mm over distances of less than 100 km [23]. In response, the natural vegetation varies from sclerophylous vegetation types such as *Eucalyptus* and *Melaleuca* woodlands on the drier margins and slopes to tall, closed canopy tropical rainforests in the wetter areas. While dramatic rainfall, topographical, and edaphic gradients have produced a highly varied mosaic of vegetation types [24] this diversity has been amplified by clearing for agriculture. Clearing focused on the prime agricultural land on the coastal plains and the inland tablelands resulting in a loss of ∼25% of forest cover [25]. This has left a mosaic of intensive agriculture (sugar, bananas and grazing) immediately adjacent to extensive areas of natural vegetation (Figure 1). Within these agricultural areas are remnant riparian and fragments of natural vegetation including rainforest, woodlands and wetlands (Laurance and Goosem 2008). This complex landscape structure provides feral pigs with a variety of landscape contexts in which to reside.

### Sample Collection and Stable Isotope Analyses

Trained trap operators provided all the samples used in the project from feral pigs that had been euthanized as part of a commercial trapping program operating under approved Australian Standard Operating Procedures for feral pig trapping. A total of 41 feral pigs (15 males, 27 females) were captured during 2010 by trapping and hunting in various locations identified as ‘rainforest’ (24 individuals), ‘sugarcane’ (10 individuals), and ‘interface’ (7 individuals) (Figure 1). Note that ‘interface’ indicates that a pig was captured in a location that has both indigenous forest and sugarcane plantations accessible, and is not a unique habitat. A total of 16 traps were used. Traps were constructed out of REO-Mesh (concrete reinforced mesh), star pickets and gates and held trapped animals in a pen. Hair was taken as close to the skin as possible, cleaned with methanol/dichloromethane (1 2 v/v) to remove surface oils and other contaminants, and the 3 mm nearest the body was sectioned for stable isotope analyses of most recent growth (basal hair). Soil was taken from seven sugarcane plantations to understand possible dietary endmember incorporation by pigs using this habitat, dried in an oven at 60°C, and homogenized prior to analysis. The homogenized skin of a single specimen of the snake, the taipan (*Oxyuranus scutellatus*) was also analyzed as a representative predator in sugarcane habitat. Longer tail hairs of seven individuals were sectioned in 3 mm increments for sequential measurement of δ^13^C and δ^15^N values. Tails from larger individuals (with longer hair available for sectioning) were purposefully selected. Moreover, we ensured that individuals were acquired from each habitat type (forest, interface, sugarcane). These requirements, coupled with the difficulty of securing tails from the trappers, limited the sample size for this part of the study to seven.

δ^13^C and δ^15^N values, carbon and nitrogen weight percent (% C and % N, respectively), and C:N ratios were determined using a Costech Elemental Analyzer fitted with a zero-blank auto-sampler coupled via a ConFloIV to a ThermoFinnigan DeltaV^PLUS^ using Continuous-Flow Isotope Ratio Mass Spectrometry (EA-IRMS) at James Cook University's Cairns Analytical Unit. Stable isotope results are reported as per mil (‰) deviations from the VPDB and AIR reference standard scale for *δ* ^13^C and *δ* ^15^N values, respectively. Precisions (S.D.) on internal standards were better than ±0.1‰ and 0.2‰ for carbon and nitrogen, respectively. We used two-way Analysis of Variance (ANOVA) to look for capture location and sex differences.

### Estimation of diet

As this study is concerned with tropical rainforest and sugarcane habitat use by feral pigs, we base habitat usage solely on *δ* ^13^C values that differ between habitats by over 15‰, whereas *δ* ^15^N values are more variable. *δ* ^13^C values were converted to % C_4_ diet using −12.2‰ VPDB as the C_4_ endmember based on analyses of local sugarcane (this study), which agreed well with an earlier study of sugarcane in the region (−12.3‰±0.4) [22]. We estimated the average composition (−28.5‰ VPDB) of local forest based on a latitudinal gradient [26]. An enrichment value of +3‰ over diet was used based on well-constrained studies of various mammals [11], [12], [14], [27]. These values are considered similar to, but more robust than, the few estimates from omnivorous mammals ∼+3–4‰ [28], [29].

Hair growth rate was assumed to be similar to the forest hog (*Hylochoerus meinertzhageni*), and taken to be 0.4 mm/day to estimate temporal patterns [20]. In order to arrive at a direct estimate of diet, sequential *δ* ^13^C values were input into a 3-pool reaction progress variable tissue turnover model [30]. We choose to use the input parameters determined for equids [11], which have also been used for forest hogs [20] and elephants [14], [31]. These pools have half-lives of 0.5, 4, and 140 days with each fraction making up 0.41, 0.15, and 0.44 of the total isotope signal, respectively [11]. Unfortunately, there are only a few studies that have estimated these pools due to the logistical difficulty of the experiment [12], [27]. Nonetheless, these estimates provide similar half-lives to the equids, and the feral pig is unlikely to have vastly different half-lives due to its omnivorous nature that incorporates large amounts of vegetative material. We expect minor modification when more specific suid parameters are determined. Initial dietary *δ* ^13^C values were assumed to be equivalent to the first sample and the average *δ* ^13^C value of the hair was used to approximate the *δ* ^13^C value of the long-term pool [14], [20].

## References

[1] Campbell TA, Long DB (2009) Feral swine damage and damage management in forested ecosystems. Forest Ecology and Management 257: 2319–2326.

[2] Cowled BD, Aldenhoven J, Odeh IOA, Garrett T, Moran C, et al (2008) Feral pig population structuring in the rangelands of eastern Australia: applications for designing adaptive management units. Conservation Genetics 9: 211–224.

[3] Roemer GW, Donlan CJ, Courchamp F (2002) Golden eagles, feral pigs, and insular carnivores: How exotic species turn native predators into prey. P Natl Acad Sci USA 99: 791–796. 10.1073/pnas.012422499 PMC117384 11752396

[4] Laurance WF, Harrington GN (1997) Ecological associations of feeding sites of feral pigs in the Queensland wet tropics. Wildlife Research 24: 579–590.

[5] Mitchell J, Dorney W, Mayer R, McIlroy J (2007) Ecological impacts of feral pig diggings in north Queensland rainforests. Wildlife Research 34: 603–608.

[6] Mitchell J, Dorney W, Mayer R, McIlroy J (2007) Spatial and temporal patterns of feral pig diggings in rainforests of north Queensland. Wildlife Research 34: 597–602.

[7] Pavlov PM, Edwards EC (1995) Feral pig ecology in Cape Tribulation National Park, North Queensland, Australia. Journal of Mountain Ecology 3: 148–151.

[8] The State of Queensland DoPIaF (2008) Feral Pig Control: A practical guide to pig control in Queensland. PR08–3564.

[9] Mitchell J, Dorney W, Mayer R, McIlroy J (2009) Migration of feral pigs (*Sus scrofa*) in rainforests of north Queensland: fact or fiction? Wildlife Research 36: 110–116.

[10] Sponheimer M, Robinson T, Ayliffe L, Passey B, Roeder B, et al (2003) An experimental study of carbon-isotope fractionation between diet, hair, and feces of mammalian herbivores. Canadian Journal of Zoology 81: 871–876.

[11] Ayliffe LK, Cerling TE, Robinson T, West AG, Sponheimer M, et al (2004) Turnover of carbon isotopes in tail hair and breath CO_2_ of horses fed an isotopically varied diet. Oecologia 139: 11–22. 14730442 10.1007/s00442-003-1479-x

[12] Zazzo A, Harrison SM, Bahar B, Moloney AP, Monahan FJ, et al (2007) Experimental determination of dietary carbon turnover in bovine hair and hoof. Can J Zool 85: 1239–1248.

[13] Rubenstein DR, Hobson KA (2004) From birds to butterflies: animal movement patterns and stable isotopes. Trends Ecol Evol 19: 256–263. 16701265 10.1016/j.tree.2004.03.017

[14] Cerling TE, Wittemyer G, Rasmussen HB, Vollrath F, Cerling CE, et al (2006) Stable isotopes in elephant hair document migration patterns and diet changes. Proceedings of the National Academy of Sciences of the United States of America 103: 371. 16407164 10.1073/pnas.0509606102 PMC1326175

[15] Sponheimer M, Grant CC, De Ruiter DJ, Lee-Thorp JA, Codron DM, et al (2007) Diets of impala from Kruger National Park: evidence from stable carbon isotopes. Koedoe-African Protected Area Conservation and Science 46: 101–106.

[16] Ehleringer JR, Cerling T (2002) C_3_ and C_4_ Photosynthesis. In: Mooney HA, Gandadell JG, editors. Encyclopedia of Global Environmental Change. Chichester: John Wiley & Sons. pp 186–190.

[17] Bird MI, Pousai P (1997) Variations of δ^13^C in the surface soil organic carbon pool. Global Biogeochem Cycles 11: 313–322.

[18] Ehleringer JR, Cerling TE, Helliker BR (1997) C_4_ Photosynthesis, Atmospheric CO_2_, and Climate. Oecologia 112: 285–299. 28307475 10.1007/s004420050311

[19] West AG, Ayliffe LK, Cerling TE, Robinson TF, Karren B, et al (2004) Short-term diet changes revealed using stable carbon isotopes in horse tail hair. Functional Ecology 18: 616–624.

[20] Cerling TE, Viehl K (2004) Seasonal diet changes of the forest hog (*Hylochoerus meinertzhageni* Thomas) based on the carbon isotopic composition of hair. African Journal of Ecology 42: 88–92.

[21] Kohn MJ (2010) Carbon isotope compositions of terrestrial C_3_ plants as indicators of (paleo) ecology and (paleo) climate. Proceedings of the National Academy of Sciences 107: 19691–19695. 10.1073/pnas.1004933107 PMC2993332 21041671

[22] Spain A, LeFeuvre R (1997) Stable C and N isotope values of selected components of a tropical Australian sugarcane ecosystem. Biol. Fert. Soils 24: 118–122.

[23] Stork NE, Goosem S, Turton SM (2008) Australian rainforests in a global context. In: Stork NE, Turton SM, editors. Living in a Dynamic Tropical Forest Landscape. Oxford, UK: Blackwell Publishing. pp 4–20.

[24] Hilbert DW (2008) The dynamic forest landscape of the Wet Tropics: present, past and future. In: Stork NE, Turton SM, editors. Living in a Dynamic Tropical Forest Landscape. Oxford, UK: Blackwell. pp 107–122.

[25] Winter JW, Bell FC, Atherton IPL (1987) Rainforest clearfelling in northeastern Australia. Proc R Soc Qld 98: 41–57.

[26] Bird MI, Chivas AR, Head J (1996) A latitudinal gradient in carbon turnover times in forest soils. Nature 381: 143–146.

[27] Zazzo A, Moloney AP, Monahan FJ, Scrimgeour CM, Schmidt O (2008) Effect of age and food intake on dietary carbon turnover recorded in sheep wool. Rapid Commun Mass Sp 22: 2937–2945. 10.1002/rcm.3693 18727150

[28] Hilderbrand GV, Farley SD, Robbins CT, Hanley TA, Titus K, et al (1996) Use of stable isotopes to determine diets of living and extinct bears. Can J Zool 74: 2080–2088.

[29] Huelsemann F, Flenker U, Koehler K, Schaenzer W (2009) Effect of a controlled dietary change on carbon and nitrogen stable isotope ratios of human hair. Rapid Commu Mass Sp 23: 2448–2454. 10.1002/rcm.4039 19603471

[30] Cerling TE, Ayliffe LK, Dearing MD, Ehleringer JR, Passey BH, et al (2007) Determining biological tissue turnover using stable isotopes: the reaction progress variable. Oecologia 151: 175–189. 17186277 10.1007/s00442-006-0571-4

[31] Cerling TE, Passey BH, Ayliffe LK, Cook CS, Ehleringer JR, et al (2004) Orphans' tales: seasonal dietary changes in elephants from Tsavo National Park, Kenya. Palaeogeography, Palaeoclimatology, Palaeoecology 206: 367–376.

